# Editorial: Neurobiological and psychophysiological underpinnings of wellbeing and prosocial connectedness

**DOI:** 10.3389/fnint.2022.995909

**Published:** 2022-08-08

**Authors:** Darren J. Edwards, Hayley A. Young, Adrián Yoris

**Affiliations:** ^1^Department of Public Health, Swansea University, Swansea, United Kingdom; ^2^School of Psychology, Swansea University, Swansea, United Kingdom; ^3^Institute of Cognitive and Translational Neurosciences (INCYT), INECO-Favaloro-CONICET, Buenos Aires, Argentina

**Keywords:** vagal nerve, interoception, wellbeing, social connection, autonomic nervous system (ANS), heart rate variability (HRV), psychological flexibility

The autonomic nervous system (ANS) and associated neurobiological pathways connecting the brain and body play a central role in health and wellbeing. For example, deficient vagal-nerve-related functioning such as interoception (sensory signals originating from inside the body which are carried by the vagal nerve to the brain) and low heart rate variability (HRV) have been linked to a range of mental health conditions, including mood and anxiety disorders, developmental, and eating disorders (Paulus and Stein, 2010; Chalmers et al., 2014; Jenkinson et al., 2018; Khalsa et al., 2018). Furthermore, a key driver of mental and physical wellbeing is the capacity for social connection; an ability that starts to develop during early childhood (Skinner and Zimmer-Gembeck, 2016). However, there is a lack of normative data concerning aspects of vagal-related functioning at different developmental stages. Finally, there is a paucity of evidence relating interoception and other forms of vagal functioning with developmental disorders such as autism spectrum disorders (ASD), or alexithymia.

Given the lack of evidence in the mentioned areas, this Research Topic aimed to: (1) Identify the ways in which interoception and other forms of vagal functioning (e.g., HRV) relate to social connectedness, perspective-taking, and prosocial behavior; (2) Increase understanding of the mechanisms and developmental stages of vagal functioning in relation to prosocial behavior and social connection; (3) Identify how deficits in interoception and other forms of vagal functioning relates to deficits in communication such as in autism spectrum disorders (ASD) and alexithymia; and (4) Place emphasis on the role that interoception and other forms of vagal functioning play as mediating and moderating factors of wellbeing such as psychological flexibility.

## A summary of the article contributions

The study by Edwards and Lowe focused on the first, third, and fourth aims of this topic. It did this by exploring how deficits in interoception (ISen) were associated with lower self-as-context (SAC) and psychological flexibility for the condition alexithymia. Higher levels of alexithymia were linked to increased psychological inflexibility, lower positive affect scores, and lower interoception. SAC was found to mediate the relation between interoception and alexithymia indicating that this was a key underlying process for this condition. The study concluded by suggesting that: (1) for treating alexithymia the development of a more comprehensive definition of the condition is required; (2) interventions that are process-based should be explored for alexithymia, such as building SAC-based perspective-taking as this was a key mediator for this condition.

Another study by Owens et al. focused on aim three of the Research Topic. They explored whether the condition called sympathoexcitation (the excitation of the sympathetic component of the ANS) was associated with autism spectrum disorder (ASD). They found basal heart rate and responses to orthostatic tests revealed that ASD was related to sympathoexcitation. They also found that sympathetic vasoconstriction was impaired in individuals with ASD. These results highlight the relevance and centrality of the autonomic system in ASD and demonstrated that it can impact on quality of life for these individuals. They conclude by suggesting that combined with core ASD symptoms, these autonomic symptoms may not be apparent to health care professionals or researchers. Like the first study by Edwards and Lowe, this suggests that the ANS needs to be explored more closely in diagnosing conditions such as ASD or alexithymia.

In a third study, Porges focuses on the feelings of safety and suggests that they emerge from internal physiological states regulated by the ANS, and specifically the vagal nerve as explained by polyvagal theory. He suggests that feelings of safety reflect a fundamental process that has enabled humans to survive, through taking advantage of opportunities brought about by trusting social engagements and mitigating metabolically costly defensive reactions. Such opportunities to prosocially co-regulate and their links to feeling safe are entrenched in humanity's evolutionary phylogenetic heritage. Porges' work fits well with the first aim of this Research Topic, as it touches on how fundamental ANS processes are in making people feel safe, which helps bring about prosocial behavior and may have many positive societal applications.

In a fourth study, Brown et al. focused on the Research Topic's fourth aim, through exploring the role of parasympathetic functioning in emotional regulation and wellbeing. They found that vagally mediated HRV moderated the influence of emotional regulation strategies. They also found that HRV acted as a marker for resilience against the adverse effects of suppression. They did not, however, find any interaction between cognitive appraisal strategies and HRV. This relates well to a systematic review in the area (Pinna and Edwards, 2020), which concluded that vagal functioning is central to emotional regulation and overall wellbeing, and Brown et al.'s study findings support this conclusion.

In a fifth paper, Wilkie et al. provide an opinion article that focuses on aim four of this Research Topic, by highlighting the complex role of the vagal nerve within the subject discipline of wellbeing. They suggest that vagal functioning plays an important role in developing “self-connection” through connecting the body and brain via the vagal nerve. This interoceptive brain-body connection results in much of the psychological experiences individuals have, such as positive affect, a sense of meaning in life, emotional regulation, and psychological flexibility.

The final paper by Poli et al. focused on aim two of this Research Topic by exploring the development of feeling safe during early infancy, and proposed that polyvagal theory could be used as a psychometric tool to assess perceived safety. They recorded the N290 event related potential (ERP) of the right fusiform face area of the cortex through Electroencephalography (EEG) of infants between 5 and 12 months old, who viewed different facial expressions (such as fear, anger, happiness, etc.). They found this ERP to be higher in amplitude in response to fearful and happy faces as opposed to angry ones, but only for 5-month infants and not 12-month infants. The authors suggested that this is evidence of a sensitive period in the development of feelings of safety. They also suggest that without this development, individuals may exhibit more sympathetic nervous system related self-protective behaviors in later life. This links well with the paper by Porges which also suggested feelings of safety emerge from internal physiological states regulated by the ANS.

## Conclusions

In conclusion, this Research Topic represents six articles, which have a common theme—they all suggest that vagal functioning, measured or interpreted through basal heart rate, HRV, polyvagal theory, or interoception, has important implications for psychological outcomes. These outcomes can be wellbeing, feelings of safety, prosocial communication, the promotion of psychological flexibility, or have implications for the severity of autism or alexithymia when defective. Such findings demonstrate that the vagal nerve, and the link between body and brain more generally, has great importance to many aspects of healthy psychological functioning and overall wellbeing. As such, more effort should be made to explore these biophysiological pathways and associated deficits that lead to measurable symptoms when diagnosing and treating conditions such as autism, alexithymia, or as a broader measure for wellbeing.

## Author contributions

All authors listed have made a substantial, direct, and intellectual contribution to the work and approved it for publication.

## Conflict of interest

The authors declare that the research was conducted in the absence of any commercial or financial relationships that could be construed as a potential conflict of interest.

## Publisher's note

All claims expressed in this article are solely those of the authors and do not necessarily represent those of their affiliated organizations, or those of the publisher, the editors and the reviewers. Any product that may be evaluated in this article, or claim that may be made by its manufacturer, is not guaranteed or endorsed by the publisher.

## References

[B1] Chalmers J. A. Quintana D. S. Abbott M. J.-A. Kemp A. H. (2014). Anxiety disorders are associated with reduced heart rate variability: a meta-analysis. Front. Psychiatry 5, 80. 10.3389/fpsyt.2014.00080 25071612 PMC4092363

[B2] Jenkinson P. M. Taylor L. Laws K. R. (2018). Self-reported interoceptive deficits in eating disorders: a meta-analysis of studies using the eating disorder inventory. J. Psychosom. Res. 110, 38–45. 10.1016/j.jpsychores.2018.04.005 29764604

[B3] Khalsa S. S. Adolphs R. Cameron O. G. Critchley H. D. Davenport P. W. Feinstein J. S.. (2018). Interoception and mental health: a roadmap. Biol. Psychiatry Cogn. Neurosci. Neuroimaging 3, 501–513. 10.1016/j.bpsc.2018.04.007 29884281 PMC6054486

[B4] Paulus M. P. Stein M. B. (2010). Interoception in anxiety and depression. Brain Struct. Funct. 214, 451–463. 10.1007/s00429-010-0258-9 20490545 PMC2886901

[B5] Pinna T. Edwards D. J. (2020). A systematic review of associations between interoception, vagal tone, and emotional regulation: potential applications for mental health, wellbeing, psychological flexibility, and chronic conditions. Front. Psychol. 11, 1792. 10.3389/fpsyg.2020.01792 32849058 PMC7419655

[B6] Skinner E. A. Zimmer-Gembeck M. J. (2016). The Development of Coping: Stress, Neurophysiology, Social Relationships, and Resilience During Childhood and Adolescence. Cham: Springer.

